# To the Editors of the Medical and Physical Journal

**Published:** 1810-05

**Authors:** 


### To the Editors of the Medical and Physical Journal.

GENTLEMEN,

ALLOW me, through the medium of your Journal, to suggest two or three observations, in reply to a Correspondent of yours, who signs himself "A Lover of the Profession."

Surely, he does not merit that title if he is so decided an enemy to Medical Reform. It has long been a matter of surprise to me, that a subject so important, and intimately connected with the welfare and interests of society at large, should so long have lain dormant, and no exertions made to root out ignorance from the profession, and crush the deadly reign of empiricism. And shall we now, at this enlightened period, find men who are still willing to tolerate these abuses, and attempt to extinguish the kindling sparks of reformation?

But I sincerely hope that the feeble efforts of a few individuals opposing the proposed plan, will not in the least diminish that laudable spirit of exertion which has characterized certain members of the profession. My sincere wish is, that their endeavours may be crowned ultimately with success, and that the *abuses* at present existing in the profession, may be removed by the mild and judicious regulations of the *Senate*.

It is comparatively of little consequence to trace the origin of empiricism, or to ascertain whether, on the whole, the metropolis, or the country, suffers most by its pernicious consequences. It is sufficient to state, that evils do exist both in town and country of most alarming magnitude, and which call loudly for the interference of the Legislature. The influence of those *monsters*, water *Doctors*, is truly alarming; the most complicated legerdemain practiced by Sorcerers, is comparatively trifling to the dark and mysterious mode of operation they have recourse to on the credulous minds of their *votaries*. Worse, and still worse, perhaps, are the cheats daily practised by the Quacks of this great and enlightened capital; but there are practitioners who, though they would openly disclaim the name of empirics, and disavow the practice, yet demand the attention of the reformers; they are men who never have been regularly educated to the profession, and whose knowledge is next to nothing; but by their assurance and assumption, they impose on the minds of the credulous; lous; they suit themselves to their passions and prejudices, and naturally gain an ascendancy over them; these men, destitute of science, practising only from a knowledge of facts, commit numerous mistakes, and too many fall victims to their misguided practice. Your Correspondent says, in one part of his paper, "that it is now impossible for the ignorant man to escape detection." But I would ask him, of what avail it is to detect the man after he has maltreated his patient, or perhaps reduced him to that state from which the regular practitioner is not able to relieve him? The deed is then done, and perhaps the consequence is inevitable. I think your Correspondent may abandon all his fears, respecting an aristocracy originating from the source whence he supposes; it is not compatable with common reason to imagine, that endeavours to remedy abuses relating to the practice of medicine, and restricting the exercise of the profession to those sanctioned by regular examinations before authorized persons, is ever likely to create disturbances in the state,* when the ultimate end of the reform in agitation is the preservation of the health of his Majesty's subjects. I shall not intrude any further on the limits of your work, but leave these observations for the perusal of your Readers.

Yours, &c.

An Enemy to Empiricism in General.

* A lover of the Profession, appears to us only to have apprehended the establishment of an aristocracy *in the profession* which might be prejudicial to the public at large, as likely to be the consequence of the proposed regulations; not that they were likely to effect any change in the established ranks of society, or could create any *disturbance in the state*. Ed.

